# Quantifying uncertainties associated with reference dosimetry in an MR‐Linac

**DOI:** 10.1002/acm2.14087

**Published:** 2023-06-24

**Authors:** Viktor Iakovenko, Brian Keller, Victor N. Malkov, Arjun Sahgal, Arman Sarfehnia

**Affiliations:** ^1^ Division of Medical Physics and Engineering Department of Radiation Oncology University of Texas Southwestern Medical Center Dallas Texas USA; ^2^ Department of Radiation Oncology Sunnybrook Health Sciences Centre University of Toronto Toronto Ontario Canada; ^3^ Mayo Clinic Rochester Minnesota USA

**Keywords:** ionization chamber, magnetic field, MRgRT, MR‐Linac, reference dosimetry, uncertainties, uncertainty budget

## Abstract

**Background:**

Magnetic resonance (MR)‐guided radiation therapy provides capabilities to utilize high‐resolution and real‐time MR imaging before and during treatment, which is critical for adaptive radiotherapy. This emerging modality has been promptly adopted in the clinic settings in advance of adaptations to reference dosimetry formalism that are needed to account for the presence of strong magnetic fields. In particular, the influence of magnetic field on the uncertainty of parameters in the reference dosimetry equation needs to be determined in order to fully characterize the uncertainty budget for reference dosimetry in MR‐guided radiation therapy systems.

**Purpose:**

To identify and quantify key sources of uncertainty in the reference dosimetry of external high energy radiotherapy beams in the presence of a strong magnetic field.

**Methods:**

In the absence of a formalized Task Group report for reference dosimetry in MR‐integrated linacs, the currently suggested formalism follows the TG‐51 protocol with the addition of a quality conversion factor *k_BQ_* accounting for the effects of the magnetic field on ionization chamber response. In this work, we quantify various sources of uncertainty that impact each of the parameters in the formalism, and evaluate their overall contribution to the final dose. Measurements are done in a 1.5 T MR‐Linac (Unity, Elekta AB, Stockholm, Sweden) which integrates a 1.5 T Philips MR scanner and a 7 MVFFF linac. The responses of several reference‐class small volume ionization chambers (Exradin:A1SL, IBA:CC13, PTW:Semiflex‐3D) and Farmer type ionization chambers (Exradin:A19, IBA:FC65‐G) were evaluated throughout this process. Long‐term reproducibility and stability of beam quality, ${\mathrm{TPR}}_{10}^{20}$, was also measured with an in‐house built phantom.

**Results:**

Relative to the conventional external high energy linacs, the uncertainty on overall reference dose in MR‐linac is more significantly affected by the chamber setup: A translational displacement along ***y***‐axis of ± 3 mm results in dose variation of < |0.20| ± 0.02% (k = 1), while rotation of ± 5° in horizontal and vertical parallel planes relative to relative to the direction of magnetic field, did not exceed variation of < |0.44| ± 0.02% for all 5 ionization chambers. We measured a larger dose variation for ***xy***‐plane (horizontal) rotations (< |0.44| ± 0.02% (k = 1)) than for ***yz***‐plane (vertical) rotations (< ||0.28| ± 0.02% (k = 1)), which we associate with the gradient of *k_B,Q_* as a function of chamber orientation with respect to direction of the B_0_‐field. Uncertainty in *P*_ion_ (for two depths), *P*_pol_ (with various sub‐studies including effects of cable length, cable looping in the MRgRT bore, connector type in magnetic environment), and *P*_rp_ were determined. Combined conversion factor *k*_Q_× *k_B,Q_* was provided for two reference depths at four cardinal angle orientations. Over a two‐year period, beam quality was quite stable with ${\mathrm{TPR}}_{10}^{20}$ being 0.669 ± 0.01%. The actual magnitude of ${\mathrm{TPR}}_{10}^{20}$ was measured using identical equipment and compared between two different Elekta Unity MR‐Linacs with results agreeing to within 0.21%.

**Conclusion:**

In this work, the uncertainty of a number of parameters influencing reference dosimetry was quantified. The results of this work can be used to identify best practice guidelines for reference dosimetry in the presence of magnetic fields, and to evaluate an uncertainty budget for future reference dosimetry protocols for MR‐linac.

## 1 INTRODUCTION

Magnetic resonance‐guided radiation therapy (MRgRT) systems integrate MRI imaging technology with modern radiotherapy linear accelerators, which provides critical advantages for daily adaptive radiation therapy.^1^, ^2^ Given the presence of strong magnetic fields, current protocols for standard reference dosimetry that are applicable to conventional radiotherapy technologies may no longer be appropriate for use in MRgRT systems.^3^ One such example, and the focus of this work, is the reference dosimetry protocol TG‐51^4^ and its addendum^5^ that have been used in North America as the code of practice for reference dosimetry. In commercialized MRgRT systems, the reference conditions outlined by TG‐51 (e.g., SSD = 100 cm)^4^ cannot be met. Furthermore, the effects of magnetic fields on beam quality measurement, reference class chamber response and the location of the effective point of measurement are among other issues that need to be studied and quantified.

An emerging consensus in previous work^6^, ^7^, ^8^ agree that the original TG‐51 formalism should be modified to the following form:

(1) $$D_{W}^{Q,B}=MN_{D,w}^{60\mathrm{Co}}k_{Q}k_{B,Q},$$

where, $D_{W}^{Q,B}$ is dose to water in the desired beam quality *Q* in the presence of magnetic field B; $N_{D,w}^{60\mathrm{Co}}$ is the chamber calibration factor under reference ^60^Co beam and reference conditions; $k_{Q}$ is the ionization chamber‐specific quality conversion factor, which accounts for the change in the absorbed‐dose to water calibration factor between the user beam quality *Q* in the absence of the magnetic field and the reference ^60^Co beam quality; *k_B,Q_* is the chamber‐specific magnetic field correction factor that is a function of (and accounts for) the orientation of the chamber with respect to the magnetic field and radiation beam axes, beam quality *Q*, as well as any deviations from reference setup conditions due to MR‐linac delivery geometry; and *M* is the corrected chamber signal as defined in TG‐51^4^ and its photon addendum^5^ by:

(2) $$M=M_{\mathrm{raw}}\;P_{\mathrm{TP}}P_{\mathrm{ion}}P_{\mathrm{pol}}P_{\mathrm{elec}}P_{\mathrm{leak}}P_{\mathrm{rp}}$$

where *M*_raw_ is the reading from electrometer without any corrections; and the six correction factors account for temperature and pressure (*P*_TP_), ion recombination (*P*_ion_), polarity effects (*P*_pol_), electrometer (*P*_elec_), leakage currents (*P*_leak_), and any off‐axis variation in the intensity profile over the sensitive volume of the chamber (*P*_rp_). The full description of these correction factors can be found in TG‐51 and its addendum and are not repeated here.

The recently formed AAPM Task Group TG‐351 is leading the effort to develop guidelines for reference dosimetry in MRgRT systems. Meanwhile, early adopters have used modified formalisms for reference dosimetry as presented in Equation (1).

The aim of this work is to investigate the uncertainty of the individual parameters in Equations (1) and (2), and to present the user with a comprehensive sample uncertainty budget analysis table for reference dosimetry in MRgRT systems. To quantify uncertainties on the various parameters, we performed our measurements in an Elekta Unity MR‐Linac. We followed similar principles and format as described in the TG‐51 photon addendum, supplemented by additional components related to the positioning of the chamber inside the MR‐linac bore. The sample uncertainty budget provided in this work can be used as a template by interested parties to evaluate the total uncertainty in reference dosimetry in their own systems by assessing/validating the exact magnitude of the uncertainties given their particular treatment device/procedure.

## 2 MATERIALS AND METHODS

**FIGURE 1:**
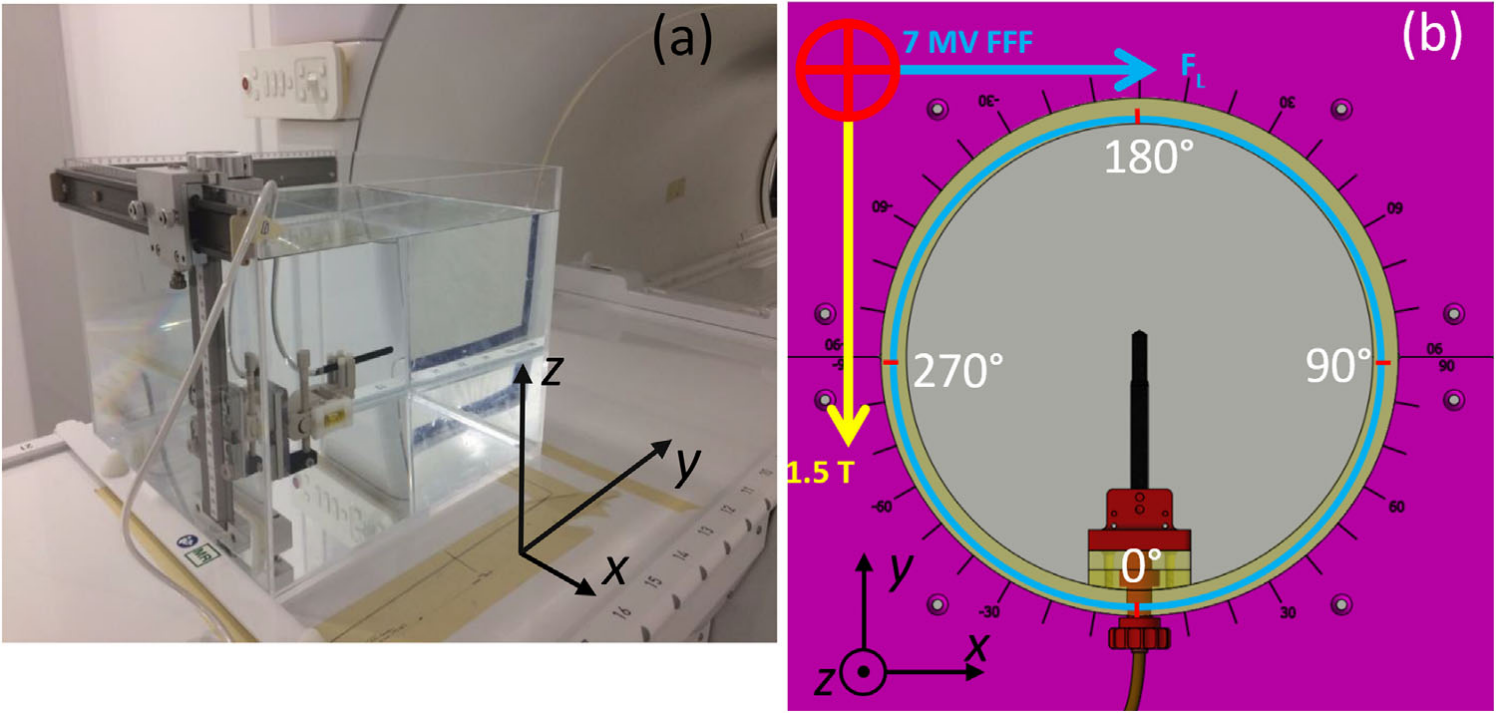
(a) In‐house built MR‐compatible water tank with A1SL chamber inside before reference dosimetry measurement. (b) Exradin A19 ionization chamber attached to the cylindrical insert (beam's eye view, Gantry = 0⁰) and positioned on the central axis of the cylinder inside the water phantom to study sensitivity to rotations in the ***xy***‐plane. The tip of the chamber defines the angle of measurement (in the example above, the chamber is located at 180⁰).

Experimental measurements were performed in an MR‐linac (Unity, Elekta AB, Stockholm, Sweden), which incorporates a 1.5 T Philips MR scanner and a 7 MV FFF beam linear accelerator with a source‐to‐axis distance (SAD) of 143.5 cm. The strong magnetic field points out of the bore towards the patient's feet. The linac output was calibrated (and specified at the isocentre) with an SAD setup and at a gantry angle of 90 degree (along ***x***‐axis as shown in Figure 1a) such that 1 monitor unit (MU) would result in an absorbed dose to water of 1 cGy for a field size of 10 × 10 cm^2^ at a depth of 10 cm in water.

### 2.1 Positioning uncertainty

To quantify the impact of positioning uncertainty on overall reference dose in the presence of a strong magnetic field, we looked at chamber response sensitivity to displacements in and out of the MR‐linac bore (along ***y***‐axis, i.e., parallel to magnetic field direction) and rotations in vertical (***yz***) and horizontal (***xz***) planes. The measurements were performed in a custom‐made acrylic (density ρ = 1.18 g/cm^3^) MR‐compatible water tank (28 × 28 × 35 cm^3^) with a 7 mm wall thickness, Figure 1a. Chambers were attached to chamber‐specific 3D‐printed holders. To study sensitivity to rotational displacement, an acrylic cylindrical insert (r = 25 cm) with 2 cm wall thickness and equipped with the same chamber mounting system was introduced in the water tank, Figure 1b. Measurements were performed at water equivalent depth 10 g/cm^2^.

Figure 1b also outlines the setup geometry convention used in this study: The chamber orientation is identified by the angle formed between the chamber tip direction and the magnetic field vector. As defined on Figure 1b: ||(0°)—chamber parallel to 1.5T magnetic field; ┴ (90°)—chamber perpendicular to magnetic field and parallel to the Lorentz force; ||(180°)—chamber antiparallel to magnetic field; and ┴ (270°)—chamber perpendicular to magnetic field and antiparallel to the Lorentz force.

Measurements were performed for 7 chambers of 5 different types: Exradin A19, Exradin A1SL (two chambers), IBA FC65‐G, IBA CC13, Semiflex 3D 31021 (two chambers). Duplicate chambers for A1SL and Semiflex 3D were used to confirm consistency of chamber response relative to the angle with magnetic field direction. Prior to any measurements, chambers were pre‐irradiated to an average dose of 20 Gy and left to stabilize for at least 5 min or when a leakage of less than 5 pC/min was obtained. Following any polarity change, the same average dose was delivered and similar stabilizing time was maintained. A PTW UE electrometer with a polarization voltage of 300 V applied to the central electrode with respect to the wall of the chamber was used to readout the charge. At least three consecutive measurements were performed per each data point or when standard deviation was < 0.1%, and the average was calculated to represent the final value of a data point.

**FIGURE 2:**
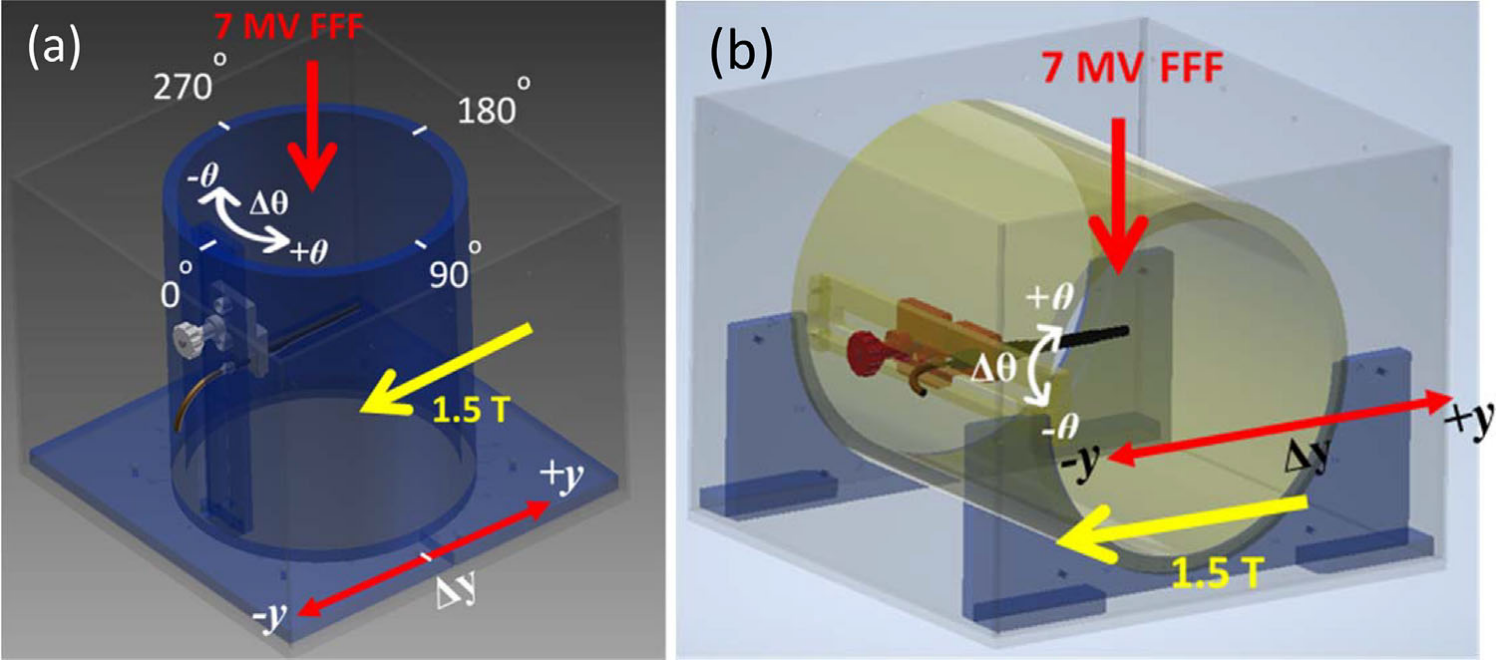
(a) CAD design of experimental setup—cylindrical insert to measure chamber's sensitivity to rotations in ***xy***–plane and displacements along magnetic field (***y***–axis) for Gantry = 0° and 90°. (b) CAD design of experimental setup—cylindrical insert to measure chamber's sensitivity to rotations in ***yz***–plane (tilt) for Gantry = 0° and 90°.

The setup geometry shown in Figure 1b and positioning verification of ionization chambers were described in our previous work^9^ that quantified ion chambers’ angular response in an MR‐linac beam. In this work, we use the same setup to measure sensitivity of chamber response to small displacements at ||(180°) geometry in order to quantify the setup uncertainty component. The ||(180°) orientation was studied because the chamber response variations are least sensitive to the magnetic field in this orientation, making it an ideal choice for reference dosimetry in the Elekta Unity MR‐Linac.^9^ To that end, the center the sensitive volume of the chamber was initially positioned at the isocenter of the MR‐linac and water equivalent depth 10 g/cm^2^, and verified with the on‐board EPID imaging. Deliberate longitudinal translations (**Δy**) ranging from ‐ 5  to +5 mm for Farmer‐type chambers and −3  to +3 mm for smaller‐volume chambers were introduced, Figure 2a. An MR‐linac patient positioning system with a precision of 0.3 mm^10^ was used to make the shifts, which were also verified with on‐board EPID imaging (pixel resolution of 0.2163 mm at isocenter). Chamber response at different **Δy** was normalized to the original (unshifted) chamber reading for analysis purposes.

To study the sensitivity of dose measurement to rotational accuracy, a similar methodology was undertaken where deliberate rotations (**Δθ**) in the ***xy***–plane, Figure 2a, were manually introduced for all chambers at increments of −5°; −2°; +2°; +5° with respect to the original (unrotated) position. Accurate engraved markings in the tank were used to achieve angular positioning of the chamber to within 0.5° accuracy. Chamber response at rotated positions were normalized to the original (unrotated) chamber reading.

Sensitivity to rotations in ***yz***–plane (tilt) was measured by repositioning the cylindrical insert in water tank in such a way that axis of the cylinder was parallel to the ***x***–axis of MR‐linac coordinate system, Figure 2b.

It should be noted that for some of the sensitivity studies, only a subset of the chambers noted above were measured. The two reference class Exradin A19 and A1SL ion chambers were purchased in‐house and were included in all sensitivity studies, however, some chambers were on loan from external institutions and were unavailable for some of the measurements. The results of the reference class A19 and A1SL chambers, with collecting volumes of 0.63 and 0.057 cm^3^, respectively, typify the relative‐response behavior of reference class chambers over a large range of collecting volumes.

### 2.2 Correction factors

We have also quantified the effects of a 1.5T constant magnetic field on various correction factors involved in the reference dosimetry protocol as shown in Equation (2). The studies parameters were:

- ***P*_TP_**. It was noted that the MRI bore fan at maximum speed impacted temperature stability of the water in the tank. To quantify the effect, the water temperature was measured every 30 min with a calibrated traceable thermometer over several 8‐h long sessions.
- ***P*_ion_** and ***P*_pol._** To assess the effect of ion recombination and polarity correction factors for the various chambers, the measurements were performed at two typical calibration depths (5 and 10 cm) and gantry angles (0° and 90°) using methods recommended in the original TG‐51 report.^4^
- ***P*_rp._** We have evaluated the uncertainty of this parameter by analyzing reproducibility of the in‐line and cross‐line profiles on the high resolution Gafchromic EBT3 films taken in the separate measurements in solid water over an area equivalent to the sensitive volume of the chamber. The films were exposed with a 10×10 cm^2^ field size at the isocenter (SAD = 143.5 cm) of the MR‐linac. To avoid air gaps, a thin layer of water (∼0.5 mm) was sprayed above and below the films prior to placement inside the phantom. To visually verify the absence of air bubbles, the films were covered on the top by a transparent slab of a 2.5 cm thick Plexiglas slab; an additional 7.5 cm of solid water was added on top of the slab to provide a total 10 cm buildup. Fourteen centimeters of solid water was also used to position the film at isocenter and provide sufficient backscatter.
- ***P*_leak._** Leakage current was measured over 2 min before every set of measurements with the linac in the “ready to deliver state,” but in the absence of radiation beam. We expect that *P*_leak_ is independent of MR‐linac systems and depends on the specific dosimetry instrumentation used.

### 2.3 Cable effects

We investigated the potential measurement sensitivity to positioning of coaxial cabling (between chamber and electrometer) under three scenarios: (i) cable passing from the conduit around an MR‐linac and to the chamber; (ii) cable passing from the conduit to the chamber through the bore of the MR‐linac in a straight line or (iii) cable passing from the conduit to the chamber through the bore but having many coil windings inside the MR–linac bore.

It is important to note that the same coaxial cable was used for all cable effect measurements with the purpose of investigating only the impact of different cable setup. The rational being that normally the same co‐axial cable drawn through the room is used for subsequent measurements. Our results therefore do not capture the uncertainty that is introduced if different cables are used.

We also quantified how an increase in the cable length impacts ***P***_pol_. Measurements were performed with and without an additional 10 m extension to the full length of the existing room cable (both the extension and original cable were of the same type).

### 2.4 Field size setting

Collimation uncertainty on reference dosimetry was quantified by varying jaw setting by 1 mm in each direction, producing three different field sizes: 9.8 × 9.8 , 10 × 10, 10.2 × 10.2 cm^2^. EPID was used to ensure the field size accuracy for every measurement.

### 2.5 Beam quality specifier, ${\mathrm{TPR}}_{10}^{20}$

**FIGURE 3:**
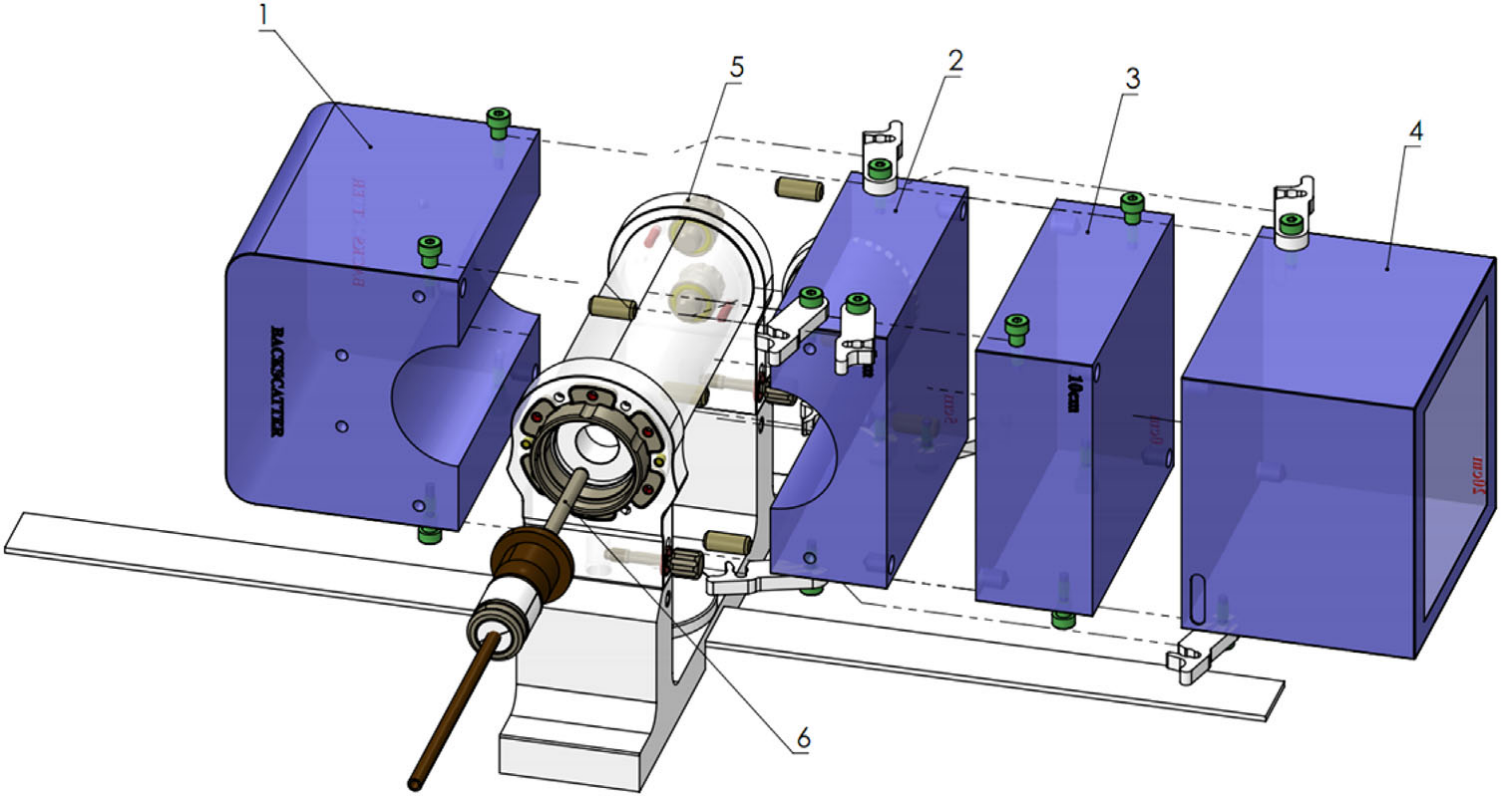
CAD images of an in‐house built QA device to measure beam quality specifier ${\mathrm{TPR}}_{10}^{20}$. Measurement setup: Gantry = 90°, 10×10 cm^2^ field size and SAD = 143.5 cm. **1** – backscatter block. **2** – 5 cm block. **3** – 5 cm block. **4** – 10 cm block. **5** – water phantom. **6** – A1SL chamber.

The presence of a magnetic field and extended SSD limits utilization of %*dd*(10)_x_ beam quality specifier, however a conversion from ${\mathrm{TPR}}_{10}^{20}$ to %*dd*(10)_x_ can be used to maintain conformance to TG‐51. This was investigated by O'Brien^6^ and Malkov and Rogers,^7^ and both groups suggest the use of a functional relationship between ${\mathrm{TPR}}_{10}^{20}$ and %*dd*(10)_x_ introduced by Kalach and Rogers.^11^ In this work, we have analyzed ${\mathrm{TPR}}_{10}^{20}$ stability over 2 years of monthly quality assurance (QA) measurements performed on our clinical Elekta Unity MR‐Linac. An in‐house phantom was built at the time of commissioning to measure ${\mathrm{TPR}}_{10}^{20}$ consistency. The phantom is indexed to the couch with a positional accuracy of 0.3 mm using the vendor‐supplied MR‐compatible index bars. Our device consists of a small cylindrical water phantom housing an A1SL chamber at a depth of 2.5 cm. Four additional acrylic blocks can be mounted onto the phantom that provide the ability to measure dose at isocenter at various total (cylinder + block) depths: 5 , 10, and 20 cm (Figure 3), while also providing sufficient backscatter. Monthly QA measurements were performed with a 10×10 cm^2^ field size at 90° calibration gantry angle, and with the chamber's central axis positioned at MR‐linac isocenter. Using the same device, ${\mathrm{TPR}}_{10}^{20}$ of another MR‐linac facility was also measured (unlike our system, the calibration depth for the other MR‐linac was at 5 cm depth).

## 3 RESULTS

### 3.1 Positioning uncertainty

**FIGURE 4:**
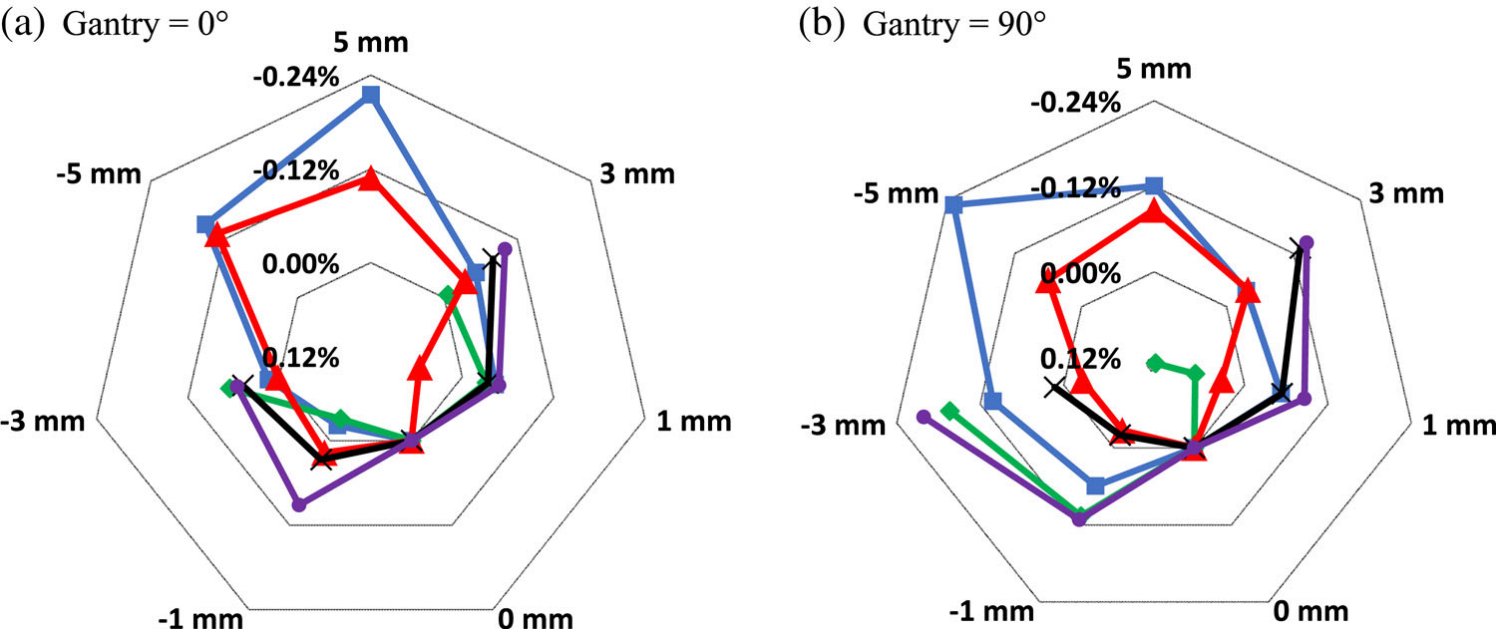
Chamber response variation as a function of displacement of chamber's centroid from the isocentre. Positive value of displacement corresponds to shifts inside the bore, negative—out of the bore. Measurement performed for chamber positioned at ||(180°), as defined on Figure 1b, 10 × 10 cm^2^ field size and two different angles of the incident beam: (a) Gantry = 0°; (b) Gantry = 90°. 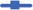 A19. 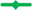
A1SL. 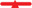
FC65‐G. 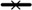
CC13. 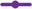
Semiflex 3D.

Displacements of up to 3 mm were measured for three of the chambers, while for the Exradin A19 and A1SL chambers, displacements of up to 5 mm were measured. The results are shown in Figure 4 in polar coordinates (radar plot), where colored lines indicate different chamber types, vertices on the plot note displacement amount in mm, and radar circular isolines indicates the variation in chamber's signal in percent relative to the unshifted value (0 mm). It is therefore clear that at 0 mm of shift, there is 0% discrepancy and all curves align. Figure 4 data is for the anti‐parallel, ||(180°), chamber alignment which corresponds to the orientation used for absolute calibration.

**FIGURE 5:**
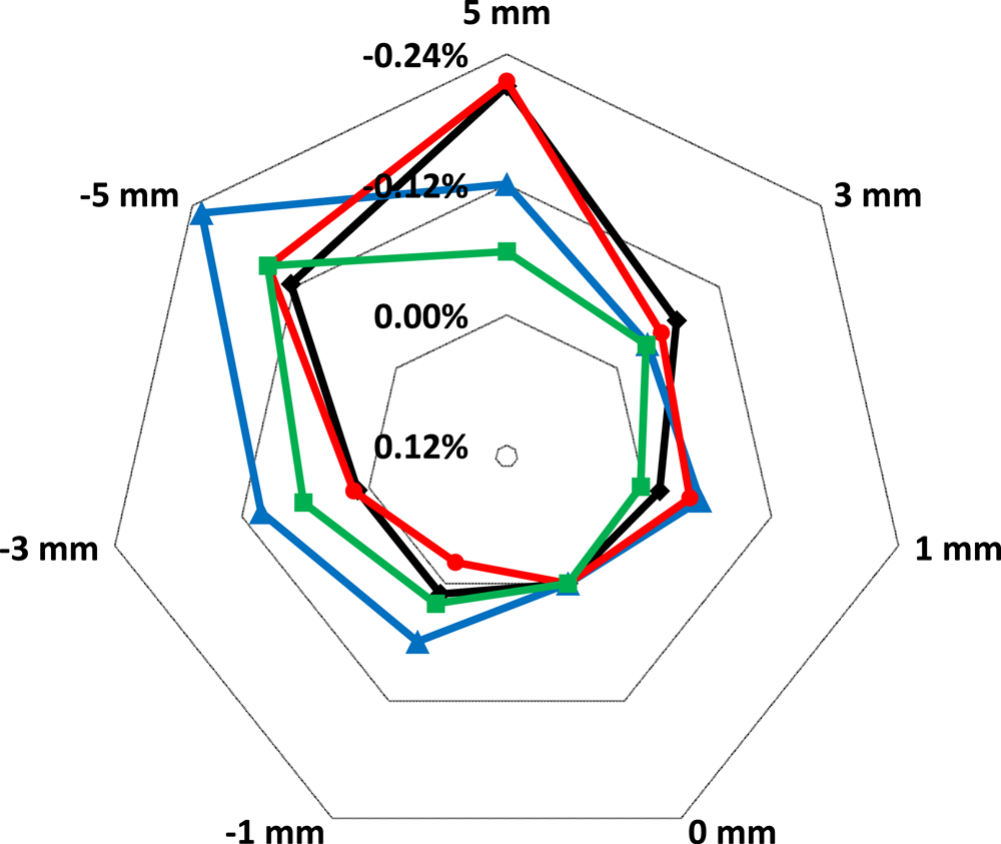
Variation of A19 response relative to the original location (centroid in the isocentre) at four different orientations of the chamber relative to magnetic field. Measurement setup: Gantry = 0°; 10 × 10 cm^2^ field size. 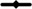
|| (0°). 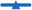
┴ (90°). 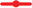
|| (180°). 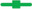
┴ (270°).

To quantify the dependence of chamber alignment with respect to the magnetic field, the positioning uncertainty (up to 5 mm in each direction) for the Exradin A19 at different chamber orientations is presented in Figure 5. The differences presented are with respect to the 0 mm displacement for each orientation.

**FIGURE 6:**
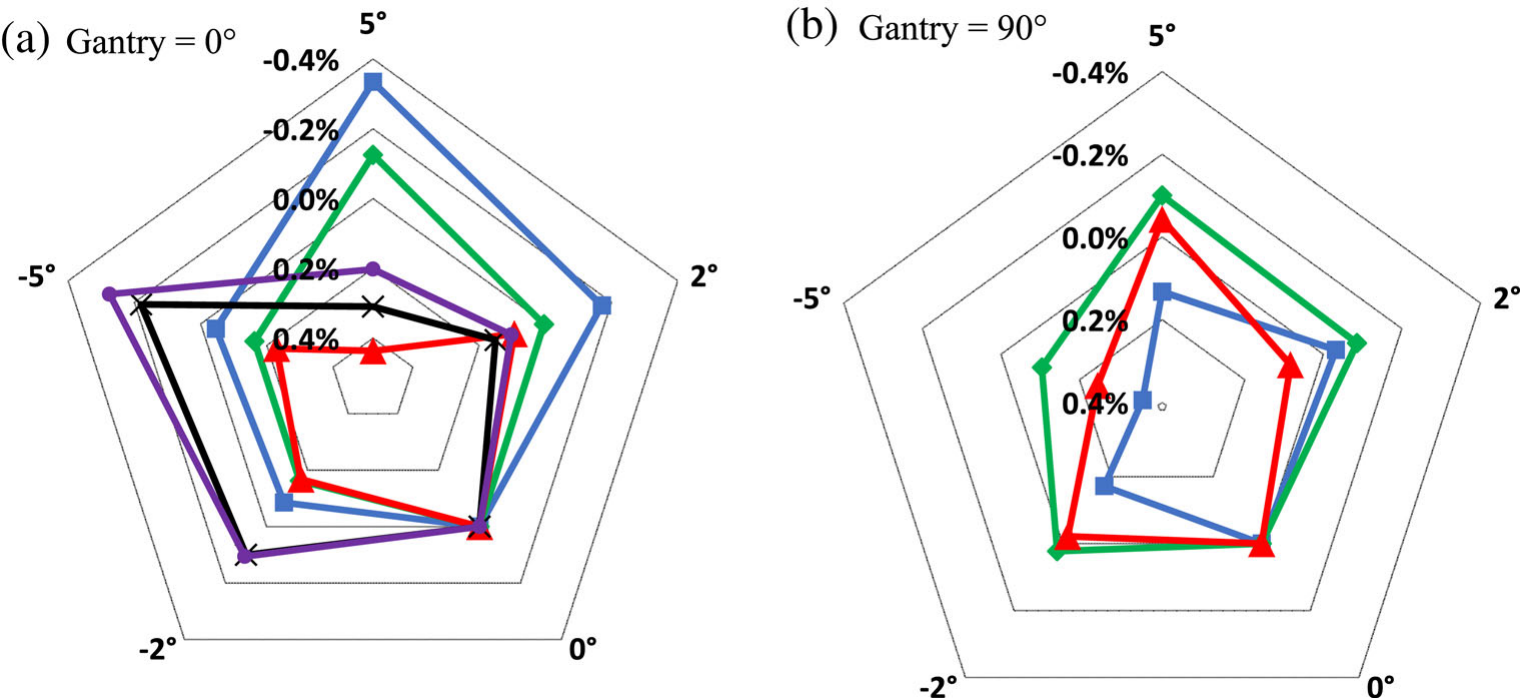
Variation of chamber's response as a function of small rotation increments in ***xy*** (horizontal) plane as defined on Figure 2a. Measurement setup: chamber positioned at ||(180°), field size 10 × 10 cm^2^ and two different angles of the incident beam: (a) Gantry = 0°; (b) Gantry = 90°. (a). 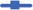 A19. 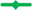
A1SL. 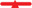
FC65‐G. 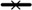
CC13. 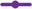
Semiflex 3D. (b). 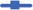 A19. 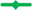
A1SL. 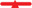
FC65‐G.

**FIGURE 7:**
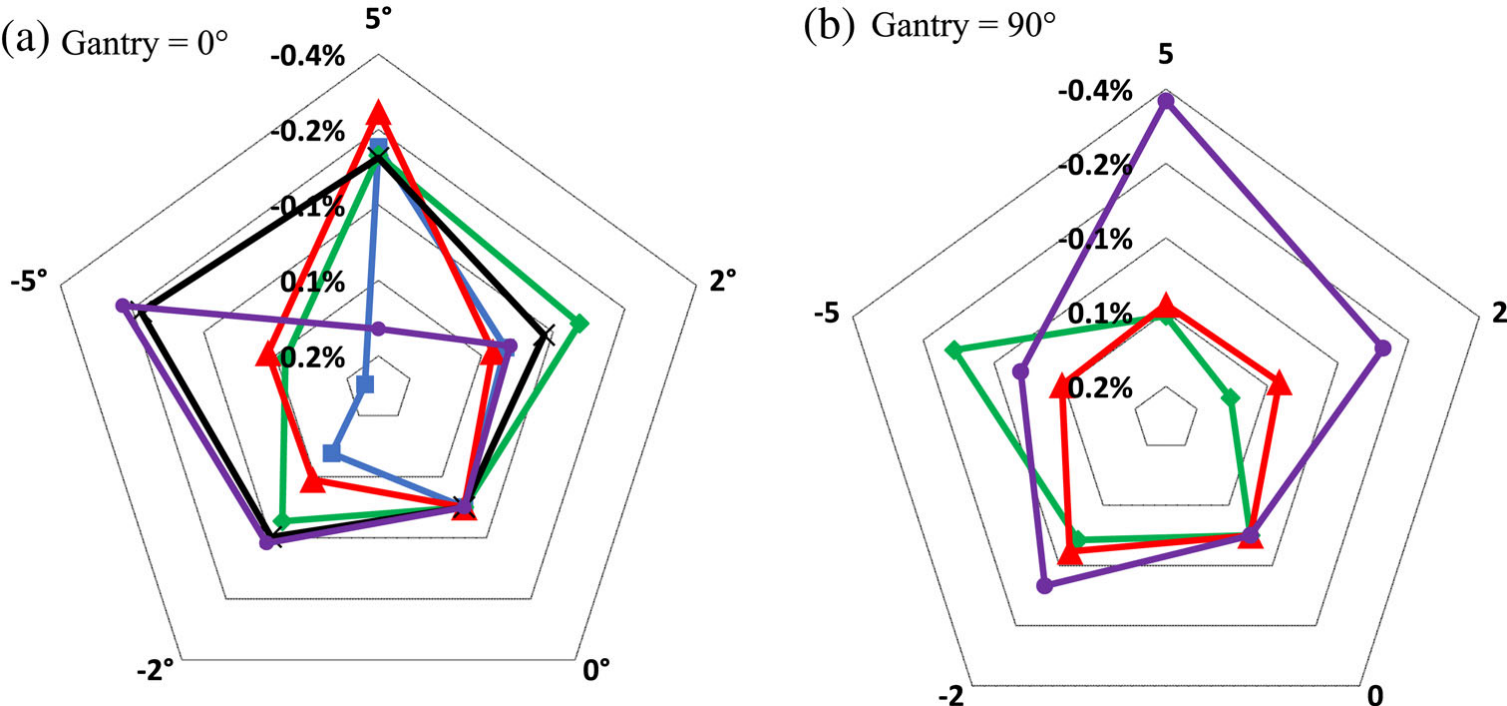
Variation of chamber's response as a function of small rotation increments in ***yz*** (vertical) plane as defined on Figure 2b. Measurement setup: chamber positioned at ||(180°),field size 10 × 10 cm^2^ and two different angles of the incident beam: (a) Gantry = 0°; (b) Gantry = 90°. (a). 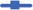 A19. 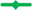
A1SL. 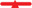
FC65‐G. 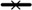
CC13. 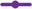
Semiflex 3D. (b). 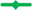
A1SL. 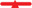
FC65‐G. 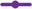
Semiflex 3D.

Sensitivity of chamber's response to rotations in the ***xy***‐plane, Figure 2a, are presented in Figure 6. For Gantry = 0°, variation of all 5 chambers were measured, Figure 6a; while for Gantry = 90°, the more commonly used Exradin A19 and A1SL, as well as FC65‐G were measured, Figure 6b. We have confirmed the overall relative response at cardinal angles (Figure 2a) for A1SL and Semiflex 3D with its duplicates. Figure 7 presents variation of chamber's response to vertical rotations as depicted in Figure 2b for the same set of chamber as in Figure 6. The choice of gantry angles (0° and 90°) is to provide data for the two commonly used gantry angles in reference dosimetry output calibration.

### 3.2 Correction factors

- ***P*_TP_**. The MR‐linac in‐bore cooling fan system, operating at maximum intensity, was determined to have a noticeable impact on the water temperature inside the calibration tank. We measured on average a 1 degree/hour cooling rate, resulting in a *P*_TP_ uncertainty of 0.17% over 30‐min data collection period if water temperature were not closely monitored. With the cooling fan system turned off, over a 6‐hour measurement session, a temperature drop of less than one degree, corresponding to an uncertainty of 0.06%, was measured. The uncertainty due to the in‐bore patient fan is in addition to the typical uncertainty associated with *P*_TP_ as described by TG‐51 addendum.
- ***P*_ion_** and ***P*_pol_** We studied the dependence of these factors as a function of chamber orientation relative to a strong magnetic field for Gantry = 0°. Table 1 presents the values of *P*_ion_ and *P*_pol_ correction factors for A1SL measured at four cardinal angles ||(0°); ┴ (90°); ||(180°); ┴ (270°) as shown in Figure 1a.
  We measured the behavior of these factors for the A1SL chamber positioned at ||(180°) and two common calibration conditions: depths (5 and 10 cm) and gantry angles (0° and 90°). Table 2 presents the values of *P*_ion_ and *P*_pol_ correction factors for A1SL measured at various depths and gantry angles.
- ***P*_rp._** Thorough analysis of the variation in dose measured on a EBT3 films over the range of the chamber size resulted in determination of a 0.03% uncertainty for *P*_rp_ for the A1SL chamber. Although an FFF beam is used in the Elekta Unity MR‐Linac system, due to the large SAD and beam attenuation through the cryostat, the beam flattens significantly at isocenter. For the A19 chamber, our estimate amounts to 0.05% from analysis of the film.
- ***P*_leak._** We found this factor to be independent of the presence of the magnetic field, thus the value used in Table 3 for this factor is the same as that found in TG‐51 addendum.

**TABLE 1:** ***P*_ion_** and ***P*_pol_** at depth = 10 cm and at four cardinal angles ||(0°); ┴ (90°); ||(180°); ┴ (270°) and beam at Gantry = 0°.

Correction factor	||(0°)	┴ (90°)	||(180°)	┴ (270°)
*P*_ion_	1.0043 (0.12%)	1.0058 (−0.03%)	1.0055 (0%)	1.0047 (0.08%)
*P*_pol_	1.0003 (−0.01%)	0.9998 (0.04%)	1.0002 (0%)	1 (0.02%)

Values in the brackets represent the difference between factors relative to ||(180°).

**TABLE 2:** ***P*_ion_** and ***P*_pol_** at two common calibration depths and gantry angles for A1SL oriented at ||(180°).

	Depth = 5 cm		Depth = 10 cm	
Gantry = 0°	||(180°)	┴ (90°)	||(180°)	┴ (90°)
*P*_ion_	1.0043 (0.12%)	1.0044 (0.11%)	1.0055 (0%)	1.0058 (−0.03%)
*P*_pol_	1.0005 (−0.03%)	1.0006 (−0.04%)	1.0002 (0%)	0.9998 (0.0%)

Values in the brackets represent the difference between factors relative to the value at ||(180°) and depth = 10 cm.

**TABLE 3:** Example of uncertainty budget for reference dosimetry performed with reference‐class equipment in an MRgRT system.

		%	
#	Component of uncertainty	Type A	Type B
*4.1*	$k_{Q}k_{B,\;Q}$ (measurement uncertainty from^13^	0.98	
*4.2*	$N_{D,w}^{60\mathrm{Co}}$		0.75
*4.3*	Depth setting	0.17	
*4.4*	Positioning (translation)	0.04	
*4.5*	Positioning (rotations)	0.05	
*4.6*	Field‐size setting		0.10
*4.7*	Charge measurement	0.23	
	Cable effect	0.04	
*4.8*	*P*_TP_		0.06
*4.9*	*P*_ion_	0.10	
	*P*_pol_	0.05	
*4.10*	*P*_leak_		0.04
*4.11*	*P*_rp_	0.03	
*4.12*	Linac stability	0.10	
	Standard error of mean	0.10	
	**Combined uncertainty {k = 1}** *User‐dependent part*	**1.29** *0.35*	

The combined estimate assumes that components in the table are uncorrelated.

### 3.3 Cable effects

We did not measure any significant difference in dosimetry based on how the tri‐axial cable is drawn, whether in a straight or coiled fashion inside or outside the bore. The differences measured were less than 0.04%, which were within the uncertainty of the measurement itself. As such, if the same coaxial cabling is used to connect the chamber and the electrometer, the way the cable is positioned with respect to the magnet does not contribute to the overall uncertainty. Moreover, we determined that different cable lengths used during measurements make no significant contribution (< 0.04%) to the uncertainty of *P*_pol_ as long as high‐quality tri‐axial cables are used.

### 3.4 Field size setting

If the jaw setting was mis‐calibrated resulting a field size that was smaller in all directions by 1 mm, that is, 9.8 × 9.8 cm^2^, the dose measured by an A1SL ion chamber would be reduced by 0.1% relative to verified 10 × 10 cm^2^. A similar value was obtained for the case where the jaws were 1 mm larger in all directions, that is, an actual field size of 10.2 × 10.2 cm^2^. Of course, these would be the extreme bounds of the problem, given mis‐calibrations are often random in nature and do not occur additively.

### 3.5 Beam quality specifier, ${\mathrm{TPR}}_{10}^{20}$

**FIGURE 8:**
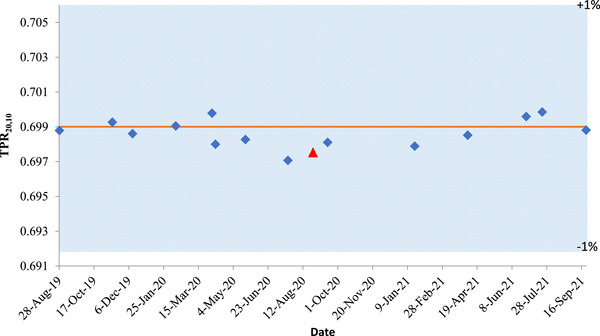
${\mathrm{TPR}}_{10}^{20}$ measured with an in‐house phantom from August 2019 to September 2021. Red triangle represents the measurement obtained at another Unity system with the same device in August 2020. 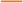 Standard. 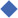 Measurement. 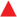
Another MR‐Linac.

Reproducibility of the ${\mathrm{TPR}}_{10}^{20}$ measured by an in‐house phantom on a bi‐monthly quality control test over a 2‐year period is shown in Figure 8. The shaded band corresponds to a normal range (± 1%) deviation from the standard value (0.699).

The standard deviation of the data points amounts to < 0.07% (k = 1). We have also measured ${\mathrm{TPR}}_{10}^{20}$ for a different MR‐linac to conclude 0.21% agreement between beam quality specifiers of the two different MRgRT systems. We have assessed the impact of uncertainty in ${\mathrm{TPR}}_{10}^{20}$ on *k_Q_* with formula (6) from the article by Andreo et al.,^12^ assuming that factor *k_B,Q_* will not be impacted significantly by varying ${\mathrm{TPR}}_{10}^{20}$. Within the same MR‐linac system, standard deviation of 0.07% translates to 0.01% in *k_Q_*, while 0.21% between two different MR‐linac systems translates to only 0.02% uncertainty in *k_Q_*.

## 4 DISCUSSION

The uncertainties on the final dose measurement derived from our measurements for parameters in Equations (1) and (2) are summarized in Table 3 for the A1SL chamber orientated at ||(180°), assuming a maximum uncertainty in translational positioning of 0.3 mm and uncertainty in rotational positioning of 0.5°during setup. Where appropriate the values from TG‐51 addendum are utilized. Uncertainty budget in Table 3 is provided for sample purposes only to show the components of the uncertainty and values we determined for A1SL chamber. It is recommended for the readers to evaluate their uncertainty budget based on their equipment and methodology. If a different chamber type is used, Figures 4, 5, 6, 7 could be used to better estimate chamber‐specific positional uncertainty. The individual uncertainties in Table 3 are discussed below.

### 4.1 Combined $k_{Q}k_{B,Q}$ determination

The combined quality conversion, $k_{Q}$, and magnetic field correction, $k_{B,Q}$, factors and their uncertainties for A1SL chambers were measured in our previous work^13^ using water calorimetry. As such, the uncertainty value in Table 3 for $k_{Q}k_{B,Q}$ is taken from our previous publication where these combined factors were measured directly by D'Souza et al.^13^

### 4.2 $N_{D,w}^{60\mathrm{Co}}$ determination

The $N_{D,w}^{60\mathrm{Co}}$ uncertainty is independent of the user and is provided by the calibration laboratory. Table 3 value of uncertainty is based on uncertainties in $N_{D,w}^{60\mathrm{Co}}$ disseminated by the National Research Council of Canada and cited in the TG‐51 addendum.

### 4.3 Chamber depth

The chamber position at depth was verified by the calibrated onboard MR‐linac EPID imaging system with a 0.3 mm precision (corresponding to maximum two pixels in image), which is comparable to the value suggested in the TG‐51 photon addendum. The uncertainty for this component is from the TG‐51 photon addendum (Section 5.A.2).

### 4.4 Chamber lateral position

Positioning uncertainty due to lateral translation was assessed from Figure 4. The same 0.3 mm uncertainty in setup was assumed given the EPID‐based positioning accuracy as described previously.

### 4.5 Chamber rotation

Positioning uncertainty due to rotations was assessed from Figures 6 and 7. It is unlikely that chamber misalignments of larger than 1⁰ go unnoticed during verification imaging. As such, we assess an uncertainty of < 0.1 % for the A1SL chamber based on Figures 6 and 7.

### 4.6 Field‐size variation

Uncertainty due to field‐size settings influence was evaluated based on our measurements with deliberate displacement of each field edge by 1 mm.

### 4.7 Charge measurement and cable effect

We assumed a charge measurement uncertainty based on TG‐51 photon addendum^5^ and an additional component (0.04%) arising from uncertainty due to different cable lengths measured in this work.

### 4.8 Temperature and pressure variations

We assessed the uncertainty of measured *P*_TP_ for the scenario where the bore fan was turned off. If this condition is met, the uncertainty of this component is the same as in the TG‐51 photon addendum.

### 4.9 Recombination and polarity effects

Uncertainty for *P*_ion_ and *P*_pol_ in Table 3 is based on TG‐51 photon addendum.^5^ Measured cable effect due to different length on *P*_pol_ is not included in this component, rather included in Table 3 earlier (in “Cable effect” component of uncertainty).

### 4.10 Leakage currents

The *P*_leak_ component of uncertainty was estimated from consecutive measurements of *P*_leak_ for the same dosimetry setup in a measurement session. The value in Table 3 is from the TG‐51 photon addendum.

### 4.11 Radial profile

We assessed the uncertainty for *P*_rp_ from the film analysis, as described in the Materials and Methods section. The value in Table 3 is from the “*P*_rp_” subsection in Results.

### 4.12 Linac output stability

Linac output variability per prescribed number of monitor units is possible over the course of performing the absolute dose measurement. The Linac stability uncertainty in Table 3 is based on the TG‐51 photon addendum.^5^

Uncertainty budget in Table 3 is presented for the A1SL chamber orientated at ||(180°); calibration depth of d = 10 cm and Gantry = 0⁰. Similar uncertainty can be achieved for Gantry = 90⁰. For other calibration conditions the components of uncertainty contributions may be different.

## 5 CONCLUSION

In this work the uncertainty contribution of the pertinent parameters for MR‐linac reference dosimetry were quantified. The results of this work can be used to identify best‐practice guidelines for reference dosimetry in the presence of magnetic fields, and to evaluate an uncertainty budget for future reference dosimetry protocols for MR‐linac.

## AUTHOR CONTRIBUTIONS

All authors have made substantial contributions to the work and development of this manuscript. All authors approved the manuscript. Viktor Iakovenko: performed measurement, analysis, and wrote the manuscript. Brian Keller: participated in scientific discussions and helped with manuscript writing. Victor Malkov: participated in measurements, scientific discussions, and manuscript writing. Arjun Sahgal: participated in scientific discussions and manuscript writing. Arman Sarfehnia: supervised the study and helped with writing of the manuscript.

## CONFLICT OF INTEREST STATEMENT

Authors do not have conflict of interest to declare.
